# Reliability of dynamic causal modelling of resting‐state magnetoencephalography

**DOI:** 10.1002/hbm.26782

**Published:** 2024-07-11

**Authors:** Amirhossein Jafarian, Melek Karadag Assem, Ece Kocagoncu, Juliette H. Lanskey, Rebecca Williams, Yun‐Ju Cheng, Andrew J. Quinn, Jemma Pitt, Vanessa Raymont, Stephen Lowe, Krish D. Singh, Mark Woolrich, Anna C. Nobre, Richard N. Henson, Karl J. Friston, James B. Rowe

**Affiliations:** ^1^ MRC Cognition and Brain Sciences Unit University of Cambridge Cambridge UK; ^2^ Department of Clinical Neurosciences and Cambridge University Hospitals NHS Foundation Trust Cambridge Biomedical Campus Cambridge UK; ^3^ Lilly Corporate Center Indianapolis Indiana USA; ^4^ Oxford Centre for Human Brain Activity, Wellcome Centre for Integrative Neuroimaging, Department of Psychiatry University of Oxford Oxford UK; ^5^ Department of Psychology University of Birmingham Birmingham UK; ^6^ Department of Psychiatry University of Oxford Oxford UK; ^7^ Lilly Centre for Clinical Pharmacology Singapore Singapore; ^8^ Cardiff University Brain Research Imaging Centre, School of Psychology Cardiff University Cardiff UK; ^9^ Department of Psychology and Center for Neurocognition and Behavior, Wu Tsai Institute Yale University New Haven Connecticut USA; ^10^ Wellcome Centre for Human Neuroimaging University College London London UK

**Keywords:** dynamic causal modelling, magnetoencephalography, parametric empirical Bayes, reliability

## Abstract

This study assesses the reliability of resting‐state dynamic causal modelling (DCM) of magnetoencephalography (MEG) under conductance‐based canonical microcircuit models, in terms of both posterior parameter estimates and model evidence. We use resting‐state MEG data from two sessions, acquired 2 weeks apart, from a cohort with high between‐subject variance arising from Alzheimer's disease. Our focus is not on the effect of disease, but on the reliability of the methods (as within‐subject between‐session agreement), which is crucial for future studies of disease progression and drug intervention. To assess the reliability of first‐level DCMs, we compare model evidence associated with the covariance among subject‐specific free energies (i.e., the ‘quality’ of the models) with versus without interclass correlations. We then used parametric empirical Bayes (PEB) to investigate the differences between the inferred DCM parameter probability distributions at the between subject level. Specifically, we examined the evidence for or against parameter differences (i) within‐subject, within‐session, and between‐epochs; (ii) within‐subject between‐session; and (iii) within‐site between‐subjects, accommodating the conditional dependency among parameter estimates. We show that for data acquired close in time, and under similar circumstances, more than 95% of inferred DCM parameters are unlikely to differ, speaking to mutual predictability over sessions. Using PEB, we show a reciprocal relationship between a conventional definition of ‘reliability’ and the conditional dependency among inferred model parameters. Our analyses confirm the reliability and reproducibility of the conductance‐based DCMs for resting‐state neurophysiological data. In this respect, the implicit generative modelling is suitable for interventional and longitudinal studies of neurological and psychiatric disorders.

## Graphical abstract

- We demonstrate the reliability of dynamic causal model of resting‐state magnetoencephalography (MEG) data.
- Using parametric empirical Bayes (PEB), we confirmed the reliability of posterior probability distributions of inferred dynamic causal modelling (DCM) parameters.
- The comparison of model evidence associated with the covariance among subject‐specific DCMs' free energies with versus without interclass correlations structure indicated reliability of first‐level DCMs within‐subjects but not between‐subjects.
- PEB confirmed the expected reciprocal relationship between a conventional definition of ‘reliability’ and the conditional dependency among inferred model parameters.

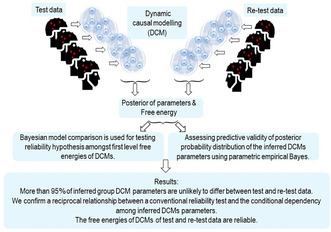

## 1 INTRODUCTION

Dynamic causal modelling (DCM) has been used widely in translational neuroscience to elucidate the underlying causes of physiological observations including electro/magnetoencephalography (MEG) (Adams et al., 2022; Adams, Hughes, et al., 2021; Gilbert et al., 2016; Jafarian, Hughes, et al., 2023; Shaw et al., 2017; Shaw et al., 2021). However, the use of imaging and analytical methods to reveal the effects of disease progression and treatment intervention rests on reliability. This article addresses the reliability of DCM, using variational Bayesian inversion of biologically informed models of neuroimaging data that can be used to characterise the neural mechanisms of cognition and the effect of disease or drugs. We test whether inferences from DCMs of resting‐state MEG data are reliable, that is, predict both the results across trials within the same session, and across separate sessions from the same participants.

DCM uses the variational Bayesian inversion of biologically motivated dynamical systems from neuroimaging data, to provide posterior estimates of unknown parameters (e.g., synaptic physiology) from a given model, and the model evidence (Friston et al., 2007; Friston et al., 2008). To compare alternative hypotheses, represented by alternative models, one uses differences in the free energy bound on [log‐] model evidence, akin to log Bayes factors (Friston, 2011; Friston et al., 2011; Jafarian et al., 2019; Kass & Raftery, 1995). Bayesian model reduction (BMR) can be used for post hoc calculation of model evidence (and posterior parameter estimates) under nested, or alternative priors. Removing redundant parameters can improve model evidence by reducing model complexity. BMR is not only computationally efficient, but also eludes local minima during model inversion (Friston & Penny, 2011). At the group level, a hierarchal Bayesian inversion known as parametric empirical Bayes (PEB) accommodates multiple first‐level (single subject) models and constrains physiological parameters according to empirical priors quantifying between subject effects (Friston et al., 2015; Friston et al., 2016; Litvak et al., 2015). PEB leverages BMR for the fast and efficient calculation of posterior estimates for each subject under group constraints. The implicit revision of empirical priors for each subject render's local minima less likely, because they are informed by the group mean. This effect is reflected quantitatively in terms of the improved (free energy bound on) model evidence at the between subjective level.

In this article, we assess the reliability of these methods in DCM, in terms of between trials and across sessions as measures of their reliability.

A classical statistical approach to measure reliability is the correlation between measures from the same participants under matched conditions (Bartko, 1966; Fisher, 1992). For two groups of data, $\left(x_{1}\left(k\right),x_{2}\left(k\right)\right)$ $\left(n=1,..k\right)$, the reliability can be defined as the modified Pearson correlation *r*, as follows:

$$r=\frac{1}{nS_{2}}\sum_{k=1}^{n}\left(x_{1}\left(k\right)-\frac{1}{2n}\sum_{k=1}^{n}\left(x_{1}\left(k\right)+x_{2}\left(k\right)\right)\right)\;\left(x_{2}\left(k\right)-\frac{1}{2n}\sum_{k=1}^{n}\left(x_{1}\left(k\right)+x_{2}\left(k\right)\right)\right)$$

(1) $$S_{2}=\begin{array}{l} \frac{1}{2n}(\sum_{k=1}^{n}{\left(x_{1}\left(k\right)-\frac{1}{2n}\sum_{k=1}^{n}\left(x_{1}\left(k\right)+x_{2}\left(k\right)\right)\right)}^{2}\\ +\;\sum_{k=1}^{n}{\left(x_{2}\left(k\right)-\frac{1}{2n}\sum_{k=1}^{n}\left(x_{1}\left(k\right)+x_{2}\left(k\right)\right)\right)}^{2}) \end{array}$$

Conventionally, a general linear model or one‐way analysis of variance (ANOVA) with random effects is used to quantify the reliability between measurements. If $y_{\mathrm{ij}}$ is the *i*th measurements in the *j* th group, ANOVA can be used to estimate the unknown group meanμ, unknown jth group random effect $\alpha_{j}$ and normally distributed random effect $\epsilon_{\mathrm{ij}}$ in the following general linear model:

(2) $$y_{\mathrm{ij}}=\mu +\alpha_{j}+\epsilon_{\mathrm{ij}}$$

The between‐group reliability is defined as $\frac{\sigma_{\alpha }}{\sigma_{\alpha }+\sigma_{\epsilon }}$, where the variance of group mean is $\sigma_{\alpha }$ and random effect variance is denoted by$\;\sigma_{\epsilon }$. However, as shown by Box and Tiao (1973) and Wang and Sun (2014), this point estimate of reliability is not always robust. Alternative reliability estimates using, for example, MCMC, can be limited by high computational burden (Mulder & Fox, 2019).

The frequentist approach to reliability considers only the expected values of parameters, that is, the maximum likelihood estimates, but not posterior variance or covariance. In ideal settings, frequentist estimates of DCM reliability may be sufficient. However, with complex models—with high posterior covariance among parameters—the reliability of the expected value of particular parameters can be very low (Frässle & Stephan, 2022; Rowe, 2010; Rowe et al., 2010; Schuyler et al., 2010). The reliability of DCM parameters has also been examined using split sampling over evoked potentials (Adams et al., 2022) and resting‐state MEG (Jafarian, Hughes, et al., 2023). However, the frequentist approach to reliability is ill‐suited to complex nonlinear dynamic systems with posterior covariance among model parameters. We therefore quantified reliability in terms of the contribution of subject or session effects using PEB; effectively, comparing between subject or session models with and without between session or subject effects. This can be interpreted as an assessment of reliability between probability distribution of parameters, in the sense that in the absence of between subject or session effects, the posterior estimates from one subject or session are similar the estimates from another.

This article has three aims: (i) to use variational Bayes to test the reliability of evidence estimates (i.e., free energy) under DCMs inverted from MEG data; (ii) to leverage the PEB to test reliability, in terms of inference about effective connectivity (i.e., posterior probability distribution of parameters) and (iii) test the influence of covariance among posterior parameters from DCM on the reliability of parameter estimates. Reliability refers to the ability to consistently reproduce a given result (and that instruments or tools used to interrogate data do so in a consistent, reproducible way). Our proposed method based on PEB (for ‘group DCM’) considers the reliability of ‘inferences’ about synaptic physiology, both within‐session and between‐session. This precludes the use of classic metrics of reliability (e.g., correlation, or intraclass correlation coefficient [ICC]), because these classical statistics can only be used with singular measurements (i.e., without uncertainly), as opposed to the probability distributions that underlie the inference (of synaptic physiology) using DCM. Reliability plays a crucial role in assessing reproducibility, generalisability, and predictive validity, whether in the context of parameter estimation or model‐based inferences.

We use repeated measures of resting‐state MEG data collected from participants in the ‘*New therapeutics in Alzheimer's disease*’ study (Lanskey et al., 2022). Our focus was not on modelling the effect of disease, but on the reliability of the ensuing estimates of synaptic efficacy. We briefly describe the participants and data, collected at baseline and 2 weeks later at rest. These data were acquired in a task‐free or resting‐state, which is suited for longitudinal studies of patients with progressive diseases. In the context of Alzheimer's disease, we consider the oscillatory dynamics of the default mode network (comprising bilateral angular gyri, medial prefrontal cortex [MPFC], and precuneus). Although medial temporal cortex is a priori associated with Alzheimer's disease, MEG is relatively insensitive to activity in this region (e.g., Hillebrand & Barnes, 2002; Piastra et al., 2021). Default mode network connectivity has been studied extensively in Alzheimer's disease and its treatment (Greicius et al., 2004; Lorenzi et al., 2011). We performed first‐level DCM to estimate synaptic parameters in the default mode regions, from the cross‐spectral density (CSD) of the MEG. We then consider the reliability of model evidence estimates, using a general linear model approach and the reliability of the DCM parameters using PEB. Finally, we discuss the potential applications and limitations of the foregoing analyses. A glossary of acronyms and variables used in this article are provided in Tables 1, 2 and 3.

**TABLE 1:** Acronyms.

Acronyms	Description
AMPA	α‐Amino‐3‐hydroxy‐5‐methyl‐4‐isoxazolepropionic acid
BMR	Bayesian model reduction
CMM	Conductance microcircuit model
DCM	Dynamic causal modelling
FT	Fourier transform
fMRI	Functional magnetic resonance imaging
GABA	Gamma‐aminobutyric acid
GLU	Glutamate
MEG	Magnetoencephalography
NMDA	N‐methyl‐d‐aspartate receptors
PEB	Parametric empirical Bayes
PSDs	Power spectral density that was derived from MEG data
ss, ss, inh, dp	Superficial pyramidal cells, spiny stellate excitatory neurons, interneurons, deep pyramidal cells

**TABLE 2:** Glossary of variables and expressions in the conductance‐based model (CMM‐NMDA).

Variable	Description
u	Exogenous input
V	Mean depolarisation of a neuronal population
$\sigma \left(v\right)$	The neuronal firing rate—A sigmoid squashing function of depolarisation
L	Lead field vector mapping from (neuronal) states to measured (electrophysiological) responses
$g_{x}\left(\omega \right),g_{o}\left(\omega \right),g_{y}\left(\omega \right)$	Spectral density of (neuronal) state fluctuations, observation noise and measurement, respectively
$\nabla_{x}f$	System Jacobian or derivative of system flow with respect to (neuronal) states
$k\left(t\right)=\text{FT}\left[K\left(\omega \right)\right]$	First‐order kernel mapping from inputs to responses; c.f., an impulse response function of time. This is the Fourier transform of the transfer function
$K\left(\omega \right)=\text{FT}\left[k\left(t\right)\right]$	The frequency transfer function modulates the power of endogenous neuronal fluctuations to produce a (cross‐spectral density) response. This is the Fourier transform of the kernel

**TABLE 3:** Parameters of the neuronal CMM‐NMDA model (see also Figure 1). The $\left(i.j\right)$ element in the matrix associated with parametrisation of intrinsic connection H, means connections that originate from population j and target population i in a region (here elements 1–4 correspond to ss, sp, inh, and dp layers, respectively). The $\left(i.j\right)$ element in the matrix associated with parametrisation of extrinsic connections A and AN, means connections that originate from population j in a region and project to population i in a distal region (here elements 1–4 correspond to ss, sp, inh, and dp layers, respectively).

	Description	Parameterisation	Prior
κ	Rate constants of ion channels, AMPA, GABA, and NMDA, respectively	$\exp\left(\theta_{\kappa }\right)\cdot \kappa$ $\kappa =\left[4,16,100\right]$	$p\left(\theta_{\kappa }\right)=N\left(0,1/16\right)$
C	Membrane capacitance of ss, sp, inh, and dp populations, respectively	$\exp\left(\theta_{c}\right)\cdot C$ $C=\left[128\;128\;256\;32\right]/1000$	$p\left(\theta_{c}\right)=N\left(0,1/16\right)$
H	Intrinsic connections	$\exp\left(\theta_{H}\right)\cdot H$ H = $\left[\begin{array}{l} 8\;0\;2\;0\\ 4\;8\;8\;0\\ 4\;0\;32\;2\\ \;0\;4\;8\;128 \end{array}\right]$	$p\left(\theta_{H}\right)=N\left(0,1/32\right)$
A	Extrinsic forward connection	$\exp\left(\theta_{A}\right)\cdot A$ A = [1 0; 0 1; 0 2; 0 0]/8	$p\left(\theta_{A}\right)=N\left(0,1/8\right)$
AN	Extrinsic backward connection	$\exp\left(\theta_{\text{AN}}\right)\cdot \text{AN}$ AN = [1 0; 0 1; 0 2; 0 0]/8	$p\left(\theta_{A}\right)=N\left(0,1/8\right)$
L	Sensor gain	L	$p\left(L\right)=N\left(1,64\right)$
J	Contribution of spiny stellate population ($J_{\mathrm{ss}}$) and deep pyramidal ($J_{\mathrm{dp}}$) to observation data	$J_{*}$, (*=dp,ss)	$p\left(J_{\mathrm{ss}}\right)=N\left(0,1/16\right)$ $p\left(J_{\mathrm{dp}}\right)=N\left(0,1/16\right)$
a	Endogenous random fluctuation with transfer function $\frac{a_{1}}{\omega^{a_{2}}}$	$\exp\left(a\right)$	$p\left(a_{1,2}\right)=N\left(0,1/128\right)$
d	Structural cosine coefficients of endogenous random fluctuation	$\exp\left(d\right)$	$p\left(d_{1,2,3,4}\right)=$ $N\left(0,1/128\right)$
b	Common sensor noise with transfer function $\frac{b_{1}}{\omega^{b_{2}}}$	$\exp\left(b\right)$	$p\left(b_{1,2}\right)=N\left(0,1/128\right)$
c	Specific sensor noise with transfer function $\frac{c_{1}}{\omega^{c_{2}}}$	$\exp\left(c\right)$	$p\left(c_{1,2}\right)=N\left(0,1/128\right)$
f	Scaling some frequencies as model of data filtration	$\exp\left(f\right)$	$p\left(f_{1,2}\right)=N\left(0,1/128\right)$
D	Neuronal delay between regions and within layers	$\exp\left(D\right).D$ $D=\left[2,16\right]$	$p\left(D\right)=N\left(0,1/64\right)$

## 2 MATERIALS AND METHODS

We tested the reliability of probability distribution of DCM parameters in terms of the evidence for between session (and subject) effects, inferred from repeated‐measures data with conductance‐based dynamic causal models.

### 2.1 Participants

The study received ethical approval from the Cambridge 2 Research Ethics Committee. Participants provided written informed consent. Participants met clinical diagnostic criteria for symptomatic Alzheimer's disease, including mild cognitive impairment, with positive amyloid biomarkers (Lanskey et al., 2022). Principal participants (fourteen) undertook two MEG scans in a resting‐state with eyes open, in two sessions that were 2 weeks apart—at a similar time of day—and without any medication changes. Their average age was 74.8 (standard deviation ±7.33), mini‐mental state examination score was 26.1/30 (standard deviation ±3.05) and Addenbrookes Cognitive Examination (revised) score 78.5/100 (standard deviation ±10.5). A second set of participants underwent baseline MEG, with closely matched age 73 (±5.33) and cognition.

#### 2.1.1 Resting‐state MEG data

Then, 5 min of resting‐state MEG data (with eyes open) were collected using an Elekta Vector View system with 204 planar gradiometers and 102 magnetometers. MEG data were recorded continuously with 1000 Hz sampling rate. Participants' horizontal and vertical eye movements were recorded using bipolar electrooculogram and electro‐cardiogram electrodes. Five head position indicator coils were placed on an EEG cap to track the head position. Three fiducial points (nasion, left, and right pre‐auricular) and >100 head shape points were digitised using Polhemus digitisation.

The Elekta Neuromag toolbox (Elekta Oy), with MaxFilter v2.2.12 was used for the detection and interpolation of bad sensors, signal space separation to remove external noise from the data and head movement correction. Then we pre‐processed the data by downsampling to 500 Hz, band‐pass filtering between 0.1 and 100 Hz, and applying a notch filter between 48–52 Hz and 98–102 Hz. We then performed artefact rejection/removal using ICA, with EOG data. We epoched the data into 1000 ms segments and repeated each epoch's artefact rejection and removal.

Using T1‐weighted structural MRI (3 T Siemens, TR = 2300 ms, TE = 2.91 ms, resolution 1 mm), we performed DICOM conversion to NII and inverse‐normalised the canonical mesh, size 2. We co‐registered the MRI to the mesh using three fiducials and head shape points to create the forward model for MEG with the single shell boundary element model method. We used ‘COH’ source inversion (Litvak et al., 2011) for extracting four default mode network sources/regions in left and right angular gyri (LAG [49 −63 33], RAG [−46 −66 30]), MPFC [−1 54 27], and Precuneus (PPC) [0 −55 32]. We used the induced source inversion option over 1000 ms epochs, frequency range 0.1–100 Hz, with fusion across magnetometers and gradiometers. We extracted principal components of power spectral densities over trials, as data features for the DCM of CSD.

We use three sets of resting‐state eyes open MEG data for the reliability study: (i) split sampled baseline data where each individual patients is divided into odd and even epochs, (ii) baseline versus 2 weeks data, and (iii) data from baseline and a second set of participants at baseline.

### 2.2 DCM of resting‐states MEG data

We use DCM for CSD, SPM12‐version 8163 (Friston et al., 2012; Moran et al., 2007; Moran et al., 2011) for inferring parameters and the marginal likelihood of the conductance‐based biophysically canonical microcircuit model (*cmm‐nmda* model in SPM12) from spectral features of MEG data. DCM for CSD explains the frequency content of MEG data in terms of a local linear perturbation (due to endogenous neuronal fluctuations) around the fixed point of a nonlinear model of canonical neuronal circuitry (Basar et al., 2012; Haken, 1977).

**FIGURE 1:**
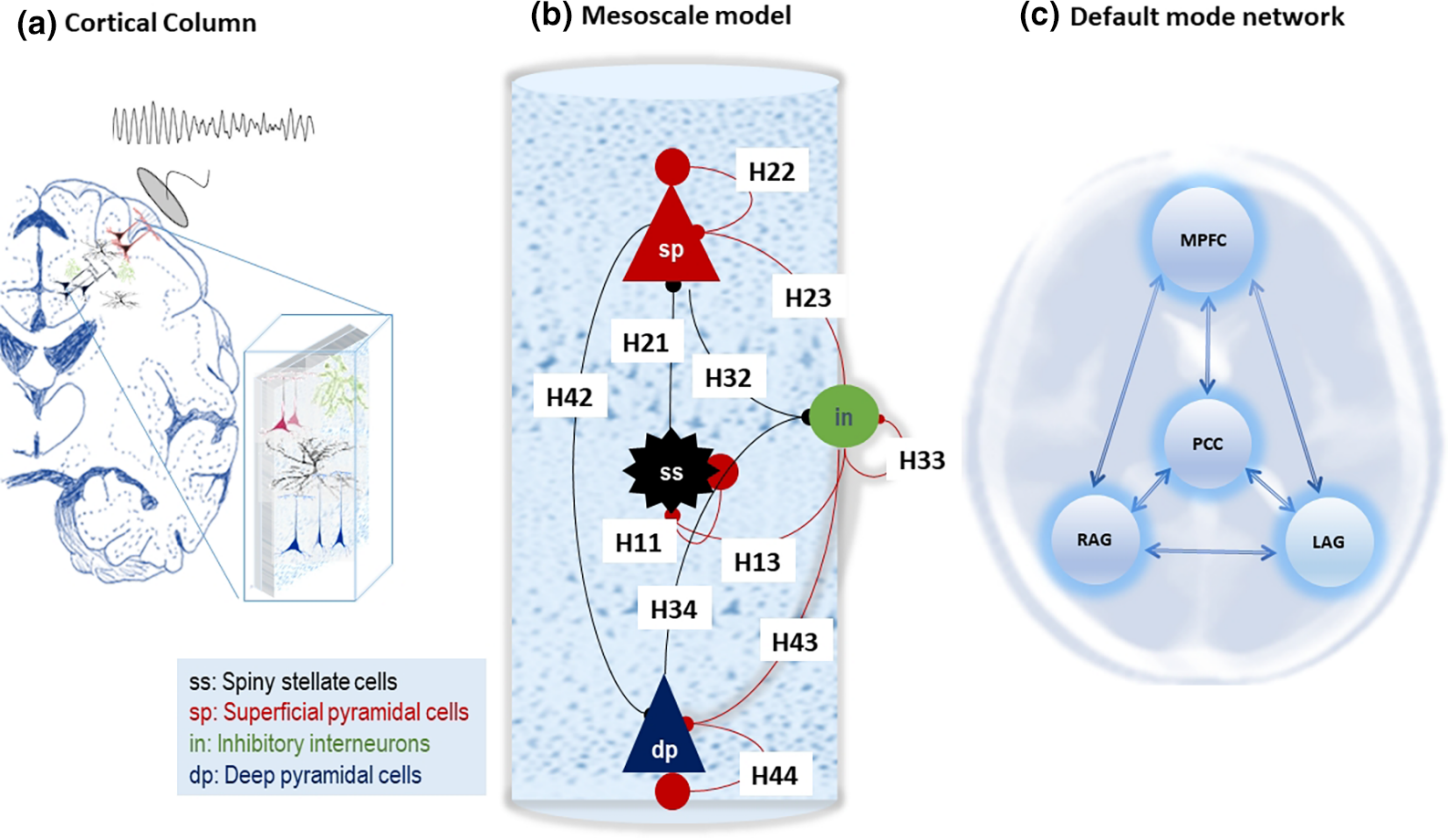
Mesoscale model of MEG data. Panel (a) illustrates a cortical column as the origin of electrical brain activity as recorded by neuroimaging modalities such as MEG. Panel (b) illustrates the laminar specific conductance based model with superficial (sp) and deep pyramidal (dp) cells in the top and bottom layers, respectively, excitatory interneurons (spiny stellate cells, ss) situated in layer four, and inhibitory interneurons that are distributed across all layers and modelled using one population. The dynamics of ion compartments with a population are governed by the Morris–Lecar model. This model explains the dynamics of different ion currents: NMDA, AMAP, and GABA and passive ion current and membrane capacitance as explained in Equation 1. Panel (c) shows the fully connected default mode network which contains MPFC. PCC, RAG, and LAG sources.

The conductance‐based model describes the electrical activity of a cortical source based on the interactions of four neuronal populations: excitatory spiny stellate cells, superficial pyramidal cells, inhibitory interneurons, and deep pyramidal cells, as shown in Figure 1. Each cortical source is connected to other regions via forward connections (that originate from the superficial pyramidal population and project to excitatory spiny stellate and deep pyramidal cells of other regions) and backward connections (that originate from deep pyramidal cells and project to superficial pyramidal and inhibitory populations in the target source). Each population is modelled by a Morris–Lecar model (driven by random endogenous fluctuations) (Moran et al., 2013) as follows:

$$\frac{\mathrm{dV}}{\mathrm{dt}}=\begin{array}{l} \frac{1}{C}[g_{L}\left(V_{L}-V\right)+g_{\text{AMPA}}\left(V_{\text{AMPA}}-V\right)+g_{\text{GABA}}\left(V_{\text{GABA}}-V\right)\\ +\;g_{\text{NMDA}}\;m\left(V\right)\left(V_{\text{NMDA}}-V\right)]+u \end{array}$$

(3) $$\frac{dg_{*}}{\mathrm{dt}}=\frac{1}{\tau_{*}}\;\left(\sum_{k=\mathrm{sp},\mathrm{inh},\mathrm{dp},\mathrm{ss}}\;S_{k}\;\sigma_{k}-g_{*}\right)+u,\;*=\left[L,\text{AMPA},\text{GABA},\text{NMDA}\right]$$

In Equation 3, the membrane potential of each population is denoted by V, the conductance of ion channels are $g_{L}$, $g_{\text{NMDA}}$, $g_{\text{AMPA}}$, and $g_{\text{GABA}}$. The input to the model is which represents random endogenous fluctuations. Constant parameters are 𝐶 as the membrane capacitance, L as a passive leak current with a fixed conductance, and $\tau_{*}$ as ion channel receptor time constants. $V_{L}$, $V_{\text{NMDA}}$, $V_{\text{AMPA}}$, and $V_{\text{GABA}}$ denote the reversal equilibrium potentials of the ion channels. The dynamics of depolarisation is equipped with activity‐dependent magnesium channels, which are modelled as $m\left(V\right)=\frac{1}{1+0.2\;\exp\;\left(-\alpha_{\text{NMDA}}V\right)}$. Afferent presynaptic firings from a population k are denoted by $\sigma_{k}$, which is scaled by connectivity $S_{k}$ which can be, in nature, excitatory AMPA, NMDA or inhibitory GABA afferent intrinsic $H_{k}$ or forward and backward extrinsic AMPA (denoted by A) and NMDA (denoted by AN) connections. Glossary of variables and prior for unknown parameters are reported in Tables 2 and 3, respectively.

The generative model of MEG data (denoted by y) can be written as a partially observed dynamical system as follows (please see Table 2 for definition of notations):

(4) $$\begin{array}{ll} \dot{x} & =f_{\theta }\left(x,u\right)\\ y & =L_{s}\left(\Gamma_{J}\left(x\right)\right)+e \end{array}$$

In Equation 4, θ denotes a lump representation of all unknown parameters (i.e., $H_{k}$, τ), x is a vector of biological states in the model, $f_{\theta }\left(x,u\right)$ is a function that is the concatenated version of the right‐hand sides of Equation 1 over all populations. In Equation 4, u is the endogenous (structured pink) noise with a cross‐spectrum, $g_{u}\left(\omega ,\theta \right)=\text{FT}\left(E\left[u\left(t\right),u\left(t-\tau \right)\right]\right)$. In the second line of Equation 4, $L_{s}$ is a parameterisation of lead field projections and sensor gain, and $\Gamma_{J}\left(x\right)$ is an operator (parameterised by unknown parameters J) that links the activity of populations (e.g., weighted sum—by J parameters—of different population responses) to MEG data (through lead field projection).

Resting‐state dynamics can be modelled as a response to endogenous random fluctuations. In this DCM, a linearised neuronal model (around a stable equilibrium point) is used to generate spectral content of the MEG data. The spectral response of the neuronal model, $g_{x}\left(\omega \right)$, can be modelled as follows:

(5) $$g_{x}\left(\omega \right)=K\left(\omega ,\theta \right).\;g_{u}\left(\omega ,\theta \right).K{\left(\omega ,\theta \right)}^{T}+g_{o}\left(\omega ,\theta \right)$$

In Equation 5, $g_{o}\left(\omega ,\theta \right)$ represents the spectrum of the observation noise, which is a sum of common and source‐specific noise and $K\left(\omega ,\theta \right)=\text{FT}\;\left(\exp\tau .\nabla_{x}f\left(x,\theta \right)\right)$ ($\nabla_{x}$ is the Jacobian) is the transfer function (input to output) of the neural model (parametrised by J). The spectral response in sensor space can also be generated by the inclusion of a forward electromagnetic model into Equation 4, which is denoted by L.M (L is the sensor gain and M is the head model), as follows:

(6) $$g_{y}\left(\omega \right)=L.\;M.\;g_{x}\left(\omega ,\theta \right).M^{T}.L^{T}+\epsilon$$

In Equation 6, $g_{y}\left(\omega \right)$ is the cross spectra of the MEG data, and $\epsilon \sim N\left(0,\sigma^{2}\right)$ is a random effect (with unknown covariance). Because we perform the DCM in the source space, the forward electromagnetic model reduces to a scaling parameter.

The laminar interpretation of the conductance‐based model (e.g., superficial, deep geometry) is supported by (i) specification of priors for intrinsic connections (Table 1), (ii) the equation of the observer for MEG data, and (iii) endogenous inputs to the model which targets spiny stellate cells in layer four. This model parametrisation supports inferences about the laminar basis of degenerative brain neurological disorders (Adams et al., 2022; Adams, Hughes, et al., 2021; Adams, Pinotsis, et al., 2021; Shaw et al., 2021).

The unknown parameters in the DCM are specified as log‐scale values $\theta =\theta_{0}\exp\left(\hat{\theta }\right)$ where $\theta_{0}$ are biologically informed scaling constants for the parameter, and $\hat{\theta }$ has Gaussian distribution, with prior normal density ${\hat{\theta }}_{0}\sim N\left(0,\sum_{\theta }\right)$ of zero mean and covariance $\sum_{\theta }$. This expresses Bayesian beliefs about the range over which parameters can vary and constrains the posterior density over parameters accordingly. The prior distribution for parameters assures the stability and plausibility of the model, and we refer to them as ‘micro‐priors’ hereafter.

### 2.3 Parametric empirical Bayes

DCM at the between session or subject level implements hierarchical variational Bayesian inversion of data under empirical priors at the second level (e.g., age, disease severity, etc) on the first level (e.g., synaptic) parameters. At the first level, the neuronal model is optimised to fit each individual's data. At the second level, the estimation of some parameters is constrained by group estimates to improve group model evidence. These parameters are those that are equipped with random effects. For these parameters, there are micro‐ and macro‐priors that constrain the accompanying posterior estimates at the first and second levels, respectively.

The micro priors are taken from the physiological literature (Friston et al., 2003; Friston et al., 2015; Friston & Penny, 2011), while macro priors impose constraints on parameters based on information about the population from which the data are drawn (i.e., an empirical prior, such as age or disease severity) (Friston et al., 2016). Due to leveraging BMR, assessing the impact of macro‐scale constraints on synaptic parameters does not require re‐estimation of the first level DCMs (Friston et al., 2016; Friston, Preller, et al., 2019; Zeidman et al., 2019).

### 2.4 Reliability of DCM

#### 2.4.1 Reliability of fixed quantities without uncertainty

This section assesses the reliability between fixed effects (i.e., measurements without uncertainty or random effects), such as the free energy of a model. We denote two sets of measurements of similar phenomena by column vectors $k_{1}$ and $k_{2}$, each of which has dimension n×1 (n number of, e.g., participants) and specify the following linear model:

(7) $$k_{1}=k_{2}\;\beta +\epsilon$$

In Equation 7, β represents the intercepts of the model between the two measurements. The reliability between measurements can be specified as a covariance component of the noise term $\epsilon \sim N\left(0,\Sigma \right)$, with compound symmetry as follows (Jelenkowska, 1998; Jelenkowska, 1999).

(8) $$\Sigma =\sigma^{2}\;\left[\begin{array}{lll} 1 & \cdots & \rho \\ \vdots & \ddots & \vdots \\ \rho & \cdots & 1 \end{array}\right]$$

The covariance matrix elements are defined as $\left[\Sigma_{\mathrm{ii}}\right]=\sigma^{2}$ and $\left[\Sigma_{\mathrm{ij}}\right]=\rho \sigma^{2}$ (i≠j). The parameter ρ is the inter‐class correlation coefficient. The compound symmetry structure of a covariance matrix assumes that all off‐diagonal elements of the matrix are equal. This means that the covariance between any two variables or measurements is the same across all pairs. This is a common assumption in statistical analyses, particularly in the context of repeated‐measures or within‐subject designs. This form of covariance is relevant for measuring reliability between measurements and can be motivated in several ways. First, the compound symmetry applies the Homogeneity assumption, with equal covariances between all pairs of variables, meaning that the relationships among variables or measurements are similar across the board. This simplifies the modelling process and reduces the number of (covariance) parameters that need to be estimated. Second, there is interchangeability of observations arising from repeated measurements on the same subjects. That is, any pair of measurements are equally related to each other regardless of when they were taken. The compound symmetry assumption reflects this interchangeability by setting all off‐diagonal elements equal. Third, the compound symmetry form simplifies the covariance matrix, aiding the estimation and interpretation of the models. With fewer parameters to estimate, analyses become more efficient: this is especially relevant when dealing with limited data. In the context of our reliability analysis, the compound symmetry implies that the reliability of measurements remains consistent across different conditions or time points.

To test reliability, one can invert the general linear model in Equation 7 with and without ρ in the covariance matrix and use Bayesian model comparison to compare the respective free energies to assess the evidence for an interclass correlation. We use variational Laplace (Friston et al., 2007) as implemented in SPM12 (e.g., Friston, Diedrichsen, et al., 2019) to estimate the covariance components, with and without the interclass correlation.

#### 2.4.2 Reliability of inferred DCM parameters

To assess the reliability of parameters with random effects, we used a parametric empirical Bayesian approach to assess the contribution of random effects to conditionally dependent parameter estimates. Mathematically, let a column vector of model parameters at the first level DCM, over cohort, is $\theta_{\mathrm{np}\times 1}^{\left(1\right)}$ (n number of participants and p is the number of parameters for each participant). Then, the generative model of the PEB is given by (Friston et al., 2016):

(9) $$\begin{array}{ll} y^{\left(i\right)} & =L_{\theta^{i,1}}\left(\Gamma \left(x\right)\right)+\epsilon^{\left(i\right)}\;i=1,..,n\\ \theta^{\left(1\right)} & =\left(X⊗I\right)\theta^{\left(2\right)}+\epsilon^{\left(2\right)}\;\theta^{\left(1\right)}=\left[\theta^{i,1},\ldots ,\theta^{i,n}\right] \end{array}$$

The first line of Equation 9 is the generative model of each DCM with unknown parameters inferred from neuroimaging data at the first level. The second line models (macro‐level) empirical priors that constrain parameter estimates from the first‐level DCM. In the second line of Equation 9, $X\in R^{n\times r}$ is the design matrix with r≥1 covariant. The first column of X is equal to one and reflects the group mean; generally, the rest of the column can be defined based on empirical data. The symbol ⊗ is the Kronecker product, and I is the p×p identity matrix. The random effects have a Gaussian distribution and at the second line of equation (6A), $\epsilon^{\left(2\right)}\sim N\left(0,\Pi^{\left(2\right)}\right)$ (where $\Pi^{\left(2\right)}$ is the precision matrix or inverse of covariance). The precision matrix is parameterised with a single (hyper‐precision) parameter, *γ*, as follows (Friston et al., 2016):

(10) $$\Pi^{\left(2\right)}=I_{S}⊗\left(Q_{0}+e^{-\gamma }\;Q_{1}\;\right)$$

In Equation 10, $Q_{0}\in R^{p\times p}$ is the lower bound on the precision, defined with a small positive value. The (hyper)parameter, γ, scales a precision matrix $Q_{1}\in R^{p\times p}$, which is (by default) 16 times the prior precision of the group mean (Zeidman et al., 2019): this hyper prior ensures that random effects are small compared to prior uncertainty about the parameter in question. The objective of group DCMs is to maximise the second level free energy under the PEB constraint. The inversion of group DCM (aka PEB) starts with the inversion of each data, followed by assessing the effect of empirical prior and adjusting parameters so that the free energy is maximised. As part of group DCMs inversion, BMR is used to re‐evaluate first‐level posteriors under updated second‐level parameters (Friston et al., 2016; Litvak et al., 2015). This greatly expedites inversion of group data. It is common practice to ask whether there is any difference between two cohorts. This question can be addressed using what is known as a ‘PEB of PEBs’. This analysis entails inverting second‐level models for separate cohorts that are then combined in a third level (PEB) analysis, to identify shared characteristics and differences. The aim of a ‘PEB of PEB’ analysis is to elucidate the similarities and differences between the models representing distinct cohorts, providing insights into the underlying difference between the populations under study. Motivated by the classical definition of reliability, that is, that there are no between‐session or between‐subject effects, we evaluated the evidence for PEB models with and without these effects. This constitute the predictive validity equivalent, which is useful in the context of inferred parameters (i.e., multivariate probably distribution). We perform PEB estimation for each parameter separately; in which synaptic rate constants (T), intrinsic synaptic gain (H), extrinsic connections (A and AN), state to observation parameters (L and J), and physiological inputs (a and d) are constrained by their group average (a single column matrix of ones). We employed the PEB of PEB approach, utilizing a matrix with the first column containing ones (constant term) and the second column containing zeros and ones, indicating each group, respectively. This method was used to evaluate the evidence for between‐session or between‐subject effects.

## 3 RESULTS

**FIGURE 2:**
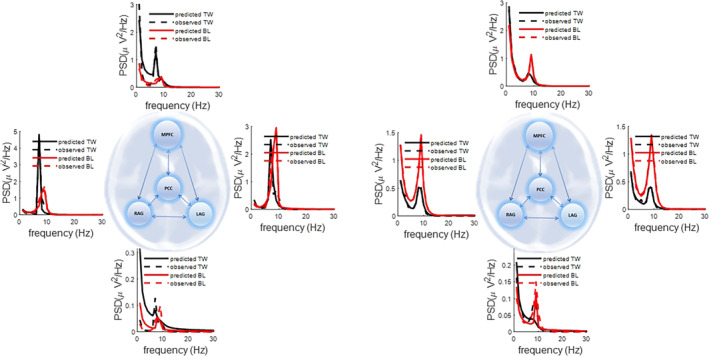
This graphics illustrate observed spectral densities (PSD) and their associated predicated response by DCM at baseline (BL) and after 2 weeks (TW) in the eyes open condition, over the four default mode network sources. The differences between baseline and 2 weeks later data are not attributable to the progression of the disease but may be linked to plasticity, psychological effects, differential fatigue, measurement noise or movement etc. Predicted DCM responses for the four‐node default model network DCMs suggest that the neuronal model replicates regions' PSDs data. Similar graphics associated with other subject's predicted and observed responses for their baseline and 2 weeks apart data are given in supplementary Figures [Link], [Link], [Link], [Link], [Link], [Link], [Link].

The power spectra of the MEG data from each source and subject are shown in the supplementary material. In addition, in Figure 2, we have shown a sample of DCM fit in terms of predicted versus observed response.

Comparison between the baseline and 2‐week spectra show that they are not identical. These differences are not attributable to the progression of the disease over such a short interval but may rise from other factors, including, for example, psychological states (e.g., fatigue), recording noise, or movements.

### 3.1 Reliability of DCM

**FIGURE 3:**
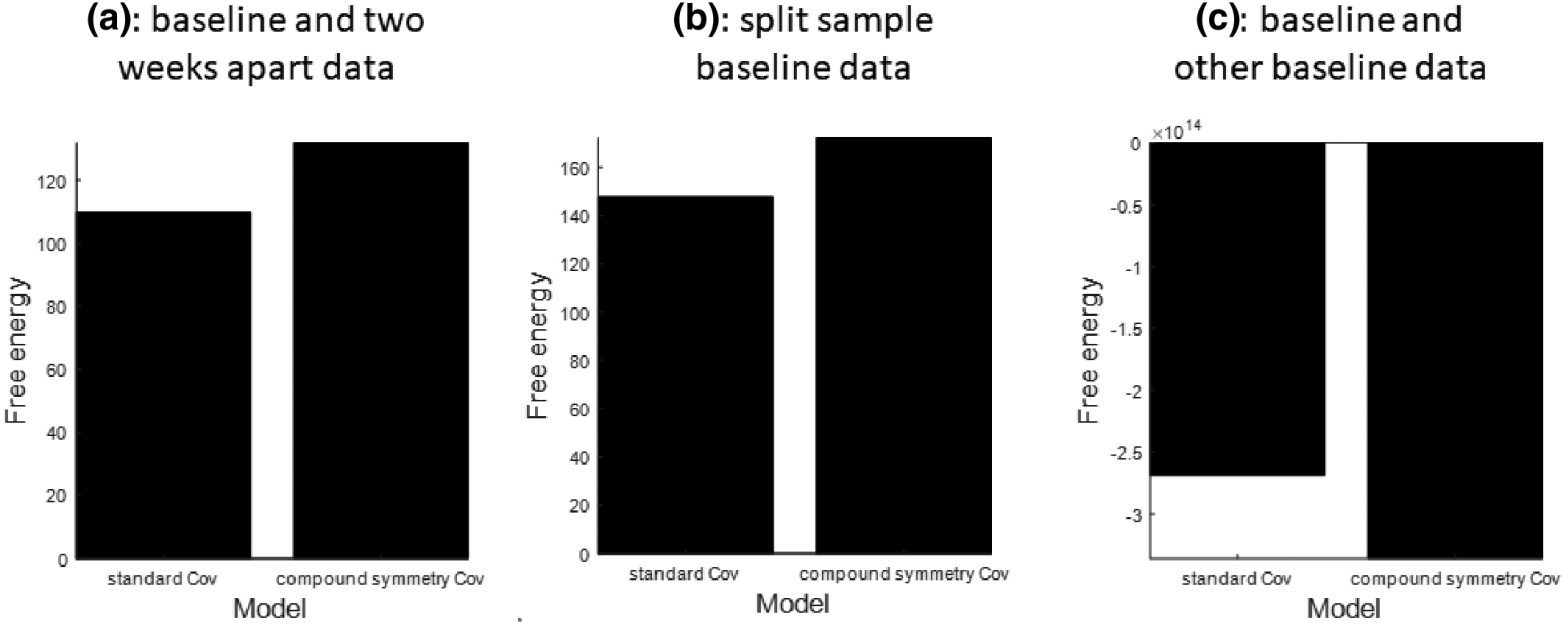
Reliability of free energies of the first level multichannel DCMs of the default mode network. There is greater evidence for the model with compound symmetry structure than the model without, when fitting data for different trials within‐subjects (panel a) or different sessions within‐subjects (panel b; baseline vs. 2‐week data), but greater evidence for the model without compound symmetry when fitting data from different subjects, as expected (panel c; baseline data).

We tested the reliability of the model's free energy estimates based on test and re‐test data: as split‐sample data from one session, or two sessions acquired in the same participants, or of two similar groups of participants, as summarised in Figure 3. The free energy estimates were highly reliable for within‐session split‐sample data, and within‐subject between session data. For the within‐subject models, the free energy was higher for the model with compound symmetry—that is, with a high interclass correlation—with a difference of 20 (equivalent to a Bayes factor of ~5 × 10^8^). On the other hand, there was no evidence for an interclass correlation between the free energy of models of data acquired from different people, even if demographically and clinically matched. This is expected: even though most of the neuronal parameters of the biophysical models that generate MEG data were similar for matched adults, differences in signal‐to‐noise and other non‐neuronal factors can have a profound effect on the free energy estimates of model evidence (a.k.a., marginal likelihood).

**FIGURE 4:**
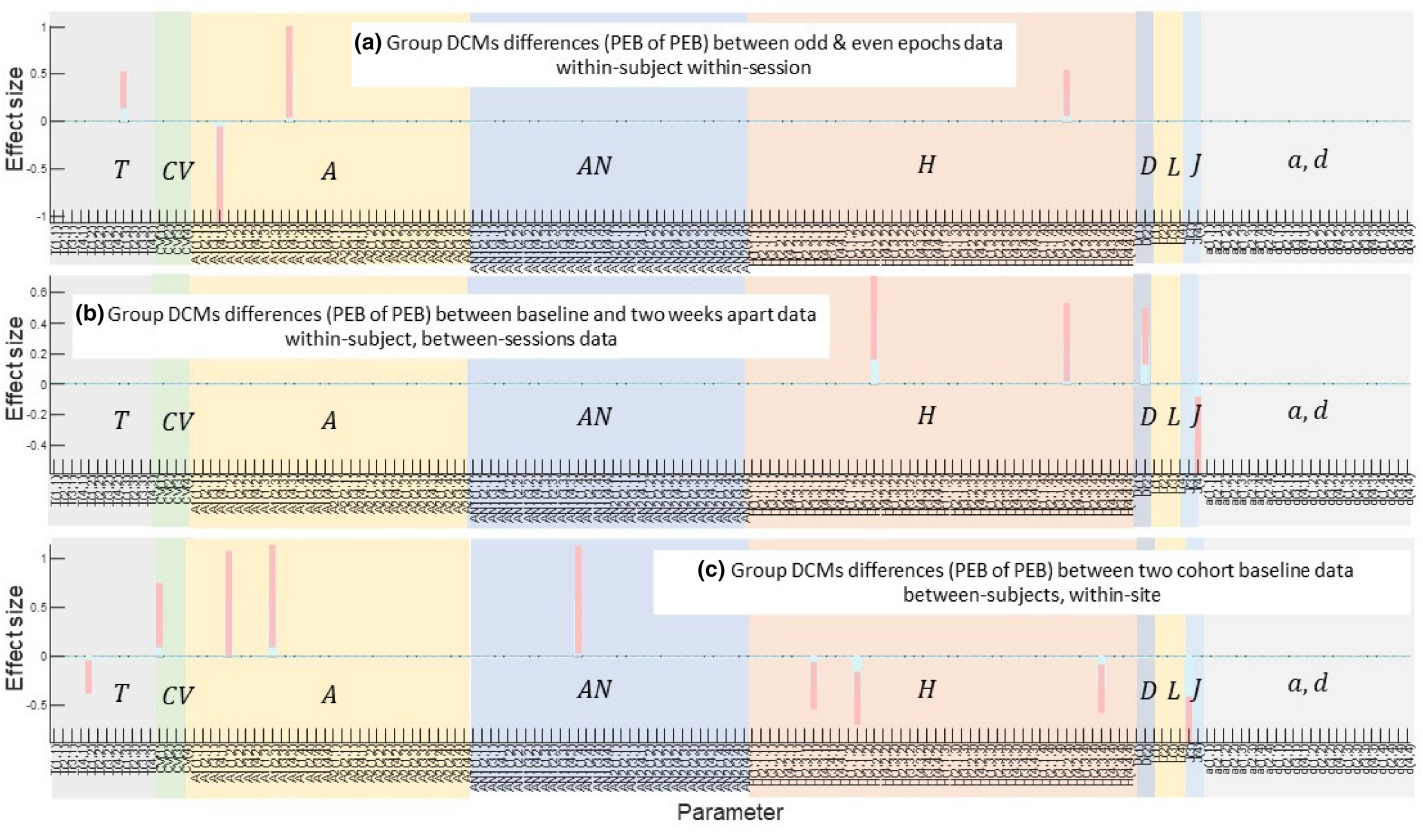
Reliability using the PEB of PEB approach for 156 inferred parameters of the fully connected default model network DCMs for (a) split sample data within‐subject within‐session, (b) within‐subject between‐sessions, 2 weeks apart and (c) between‐subjects, within‐site. Each plot illustrates the expectation of parameters (blue bar) and their 95% confidence interval (pink bar) for parameters for which there was evidence of a difference. Note that >95% of parameters do not differ within‐session, or between‐sessions. Time constants are denoted by TandCV are membrane capacitances, A and AN are between‐sources forward and backward extrinsic AMPA and NMDA connections, respectively, H are the intrinsic within‐regions connections, D are within and between regions delays, L and J are sensor gains and states that contributed to local field potentials, respectively, and a and d are the parameters of random neuronal fluctuations. The list of all 156 parameters in x axis are provided in the supplementary information.

To test for between session and subject effects, we used PEB of PEBs to assess the reliability of 156 physiological parameters; including synaptic rate constants (*T*), intrinsic synaptic gains (*H*), extrinsic forward and backward AMPA connections (denoted by A) and extrinsic forward and backward NMDA connections (denoted by *AN*), state to observation parameters (*L* and *J*), and physiological inputs (*a* and *d*). For the within‐session split‐sample analysis, the PEB of PEBs revealed that only 4 of 156 parameters differed between odd and even trials (Figure 4a). For the within‐subject between‐session analysis, the PEB of PEBs showed that only four of 156 parameters differed between sessions (Figure 4b). Finally, there was fair agreement across the two separate groups of similar participants, with nine of 156 showing evidence of between subject effects on parameter estimates (Figure 4c).

### 3.2 Reliability and the correlation of parameters

To quantify the relationship between the reliability of the posterior parameters estimates and their correlation, we examined the expected values of parameters after first level DCM and their re‐estimation by the PEB, that is, second level DCM to implement empirical priors or constraints. In Table 4, we report number of intrinsic, extrinsic forward and backward synaptic parameters that have correlations larger than 0.5 between their first‐level DCM estimates and after re‐estimation by PEB under empirical (second level) constraints. This confirms that after implementing empirical priors, their reliability improves. The PEB re‐estimates first‐level DCM parameters to improve model evidence by reducing model complexity and implicitly resolving conditional dependencies. In effect, inferred parameters become more conditionally independent, and thereby more reliable in the classical sense.

**TABLE 4:** Reliability of parameters before and after using PEB for split sampled and follow up data.

Inferred DCM parameters	Number of parameters with correlation score >0.5 in DCMs of split sample baseline data		Number of parameters with correlation score >0.5 in DCMs of 2 weeks apart data and baseline data	
	First level DCM	Re‐estimated first level DCM by PEB	First level DCM	Re‐estimated first level DCM by PEB
Intrinsic connections H	15	19	8	10
Extrinsic forward connection A	8	9	7	8
Intrinsic backward connections AN	9	10	11	12

## 4 DISCUSSION

We assessed the reliability of DCM using MEG data acquired within‐session, between‐session, and between‐subject. In all three cases, the group level conductance‐based DCM estimates, obtained from resting‐state MEG power spectra, are highly reliable in terms of the inferred neuronal parameters' distributions. To compare fixed quantities (e.g., the free energy estimates of model evidence), we used variational Laplace to estimate the covariance components due to intra‐class correlations. To assess agreement between parameters with random effects, we used ‘PEB of PEBs’ to test for between‐session and between‐subject effects, finding excellent generalisation over split‐sample and test–retest analyses. These indicate a high reliability of DCM of electrophysiological observations. In addition, we confirmed the reciprocal relationship between reliability and conditional dependency (covariance) between parameter estimates. In other words, as the covariance among parameters reduces, the reliability of their expectations increases.

The frequentist correlations of singular parameters, as typically measured by the ‘traditional’ ICC approach, are not well‐suited to the context of complex models—with high posterior covariances—such as DCM. This is primarily due to the conditional dependency between parameters. However, alternative metrics such as the free energies remain consistent between sessions, despite the presence of conditional dependency among inferred parameters. This consistency indicates that model comparison is reliable, thereby providing a useful means to specify and test different hypotheses related to session differences (e.g., a drug intervention, or disease progression). Moreover, we focus on the underlying meaning of reliability and employ the ‘PEB of PEB’ approach to determine between‐session effects. This is especially important with the use of subject‐specific biomarkers as priors in DCM, for example, in translational modelling and precision medicine. The posterior covariance between parameters is a major contributor to poor reliability (something that has been observed before but is explicitly investigated here). By improving the free energy of—that is, evidence for—the model (for instance, by using PEB as reported in Table 4), one may observe some improvement in the reliability of parameters (one can intuit this as an improvement in model evidence via reducing model complexity and, consequently, reducing the posterior correlations among parameters). By refining and providing better priors (e.g., through, e.g., MRS or PET data), the free energy of a model improves. However, it is important to note that even in the case of multimodal DCM (Jafarian et al., 2020) or pathology‐enriched DCM (e.g., Jafarian, Assem, et al., 2023) the classic reliability formulation would still not be applicable to the inferred parameters simply because the parameters are probability distributions, whereas classic reliability tests were defined for repeated singular measurements without considering uncertainty.

A key motivation for this analysis was to address the reliability of inferences from complex biophysical models in which neuronal generators are nonlinear, and parameter estimates are correlated (Litvak et al., 2019). In previous studies, individual DCM connectivity parameters have proven unreliable, except for very simple models, despite the high reliability of model evidence and model comparison for hypothesis testing (Jafarian et al., 2022; Rowe, 2010; Rowe et al., 2010; Schuyler et al., 2010). Poor reliability was attributed to their covariance, and the use of model comparison based on model evidence was recommended for hypothesis testing.

Parameter inference is sensitive to changes in the data (which may arise in repeated‐measures or between‐subject data). The translational neuronal modelling of neurological disorders presents a significant challenge, necessitating a reliable platform for hypothesis testing. DCM offers such a platform, but its utility rests on reliability for repeated measures and agreement between sessions. By utilizing the resting‐state data, we invert neuronal models of the default mode network with moderate complexity. We demonstrate that consistent conclusions can be drawn from either dataset because free energies from these data remain consistent across datasets. This complements earlier reports of the reliability of evoked responses, spectral power and functional connectivity (Colclough et al., 2016; Kumar et al., 2022; Lew et al., 2021; Marquetand et al., 2019). Indeed, the use of evoked responses rather than resting state, may increase reliability further, although resting‐state data facilitates large scale clinical studies. The reliability we demonstrate is adequate for a modelling platform for experimental medicine, improving our understanding and treatment of neurological disorders. However, using PEB (Friston et al., 2016; Friston & Penny, 2011; Litvak et al., 2015; Litvak et al., 2019) one can effectively identify the similarity of underlying neuronal dynamics between split‐sample and test‐re‐test data for ~95% of parameters; consistent with the data being drawn from the same distribution, and in our study the absence of disease progression.

Parameter estimates under nonlinear models are accompanied by a degree of co‐linearity that is, changing one parameter is equivalent to changing others. In other words, different combinations of parameters in the generative model could give rise to very similar data. The set of parameters can be considered as a manifold, which is biologically plausible (Prinz et al., 2004) but challenging when testing hypotheses based on particular parameter values. Historically, solutions to this problem have included reducing model complexity (Stephan et al., 2007), by re‐parameterisation and defining priors for the generative models for functional MRI. However, such re‐parameterisation is not straightforward, and may not be possible for generative models of M/EEG. This is because of the inherent complexity of the models of M/EEG cortical generators (Penny, 2012). To address this problem, we leveraged BMR and PEB to find nested models with lower complexity that can better capture the underlying dynamics.

The use of PEB can be seen as an extension of BMR to cohort studies. PEB is well suited to address whether model evidence from cohort data can be improved by replacing non‐informative (or weakly informative) prior parameters in DCM by empirical priors; that is, empirical constraints (Adams et al., 2022; Adams, Pinotsis, et al., 2021; Friston et al., 2022; Jafarian et al., 2021; Jafarian, Hughes, et al., 2023). In PEB, a reduced model with higher model evidence is sought at the group level by constraining parameters using group information to enhance cohort model evidence. PEB re‐estimates first‐level DCMs where all parameters are informed by the group information. If two sets of data are sampled from the same distribution, as expected within‐session, then PEB of PEB should not identify differences in the parameters of the generative model. The improvement of reliability after the application of PEB is partly due to the application of BMR during group DCM inversion, which serves to reduce the complexity and, implicitly, the posterior correlation between parameters.

The reliability of the probability distributions of parameters and model evidence from DCMs is just one aspect of their validation. It is not expected that the inferred parameters are identical, given the likely differences between the data acquired close together and in ‘similar’ conditions. Although disease progression is unlikely to a meaningful degree in 2 weeks, other differences like fatigue, anxiety, motion or scanner noise, may arise. These differences are likely to occur in addition to longitudinal or interventional ‘repeated‐measures’ studies. The effect of such cofounds for translational neuroscience is a matter of degree, and approaches to reduce the effect of such confounds include (i) using larger data samples and (ii) inclusion of informative priors on individual differences (Jafarian, Hughes, et al., 2023; Stephan et al., 2009; Zeidman et al., 2019). It is anticipated that DCM holds promise for experimental medicine studies, particularly in estimating within‐subject differences following a drug intervention or disease progression, as well as in guiding treatment decisions through model comparison. Previous studies, such as those investigating the effects of deep brain stimulation or drug interventions in Parkinson's disease and psychotic disorders (Jin et al., 2023), have demonstrated this potential application. However, some prior applications of DCM have relied on the expectation of parameters, using posterior expected values as dependent variables in repeated measures ANOVA. This approach ignores posterior uncertainty about parameter estimates, which may be essential for understanding of the underlying generators of data. One practical strategy—to address this limitation—is through BMR, which allows for the assessment of how alternative priors, such as treatment or time effects, predict and elucidate the effects of interventions on individual patients. For example, recent single‐subject work by Friston, Preller, et al. (2019) used this approach to shed light on the mechanisms of neurovascular coupling.

In summary, we have demonstrated reliable inferences based on the posterior probabilities and reliable relative model evidences, both within‐session and between‐sessions. The use of group inversion improves the free energy of first‐level DCMs and improves reliability of individual parameters. Such DCMs, including the canonical microcircuit model of MEG, provide a sufficiently reliable modelling platform to consider for use in longitudinal or interventional studies.

## AUTHOR CONTRIBUTIONS

**Amirhossein Jafarian:** Conceptualization, methodology, software, validation, formal analysis, writing original draft, data curation, visualization. **Melek Karadag Assem**: Data acquisition, pre‐processing of MEG data, review and editing. **Ece Kocagoncu**: Data acquisition, pre‐processing of MEG data, review and editing. **Juliette H. Lanskey**: Data acquisition, review. **Rebecca Williams**: Review. **Andrew J. Quinn**: Data acquisition, review and editing, **Jemma Pitt**: Data acquisition, review. **Stephen Lowe:** Review, **Vanessa Raymont**: Review. **Krish D. Singh**: Review. **Mark Woolrich**: Review. **Anna C. Nobre**: Discussion, review and editing. **Richard N. Henson**: Discussion, review and editing, **Karl J. Friston**: Conceptualization, methodology, review and editing. **James B. Rowe**: Conceptualization, methodology, review and editing, funding acquisition.

## CONFLICT OF INTEREST STATEMENT

The authors declare that there is no competing interests.

## Supporting information


**FIGURE 1S.** Power spectral densities (PSD) and their associated predicated response by DCM at baseline and after 2 weeks in the eyes open data for the subject 1 (left graphics) and 2 (right graphics).

**FIGURE 2S.** Power spectral densities (PSD) and their associated predicated response by DCM at baseline and after 2 weeks in the eyes open data for the subject 3 (left graphics) and 4 (right graphics).

**FIGURE 3S.** Power spectral densities (PSD) and their associated predicated response by DCM at baseline and after 2 weeks in the eyes open data for the subject 5 (left graphics) and 6 (right graphics).

**FIGURE 4S.** Power spectral densities (PSD) and their associated predicated response by DCM at baseline and after 2 weeks in the eyes open data for the subject 7 (left graphics) and 8 (right graphics).

**FIGURE 5S.** Power spectral densities (PSD) and their associated predicated response by DCM at baseline and after 2 weeks in the eyes open data for the subject 9 (left graphics) and 10 (right graphics).

**FIGURE 6S.** Power spectral densities (PSD) and their associated predicated response by DCM at baseline and after 2 weeks in the eyes open data for the subject 11 (left graphics) and 12 (right graphics).

**FIGURE 7S.** Power spectral densities (PSD) and their associated predicated response by DCM at baseline and after 2 weeks in the eyes open data for the subject 13 (left graphics) and 14 (right graphics).

**TABLE 1S:** The list of the parameters in order of the x axis of the Figure 4.

Supporting information.

## Data Availability

The SPM12 software (https://www.fil.ion.ucl.ac.uk/spm/) was used in this research. The code associated with this paper is available at https://github.com/NIMG-22-2183/HM-reliability-of-DCM. Minor changes were applied to the spm_ar_reml code to accommodate specifying the compound symmetry structure for the covariance of the random effect. Anonymised imaging data can be requested from the senior author, but a data transfer agreement is likely to be required under the terms of consent and data protection and the need to prevent patient identification.
