# Supplementary figures and images for: Reliability of dynamic causal modelling of resting‐state magnetoencephalography
Source: Hum Brain Mapp. 2024 Jul 11;45(10):e26782. doi: 10.1002/hbm.26782 (PMC11237883; doi:10.1002/hbm.26782)

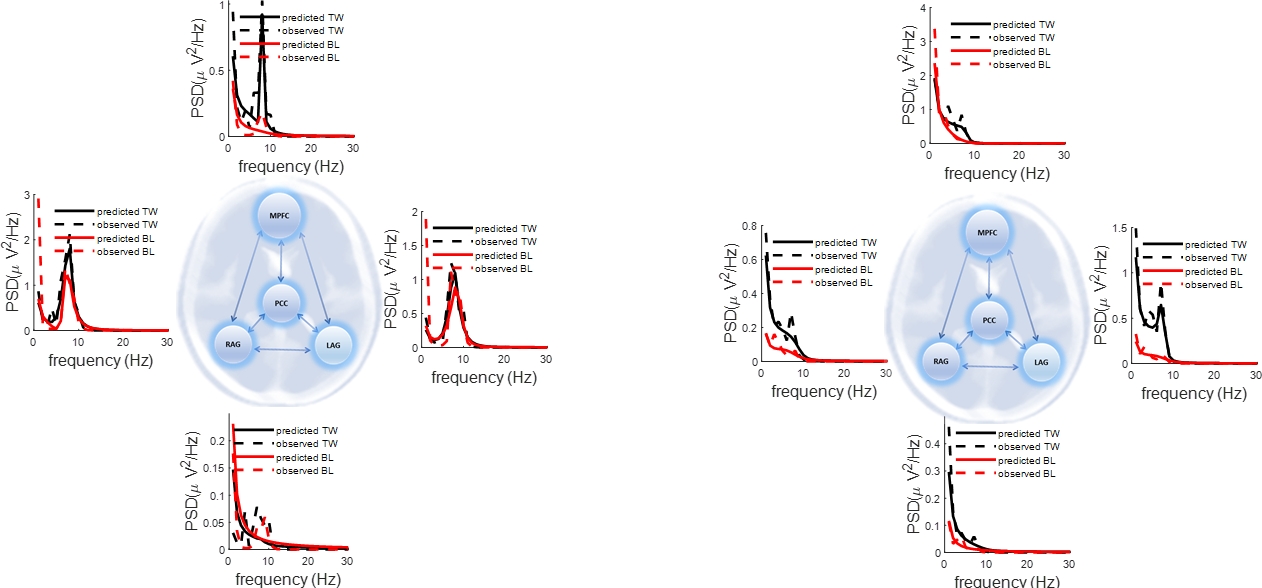

Supplement: Supplementary file 1 — FIGURE 1S. Power spectral densities (PSD) and their associated predicated response by DCM at baseline and after 2 weeks in the eyes open data for the subject 1 (left graphics) and 2 (right graphics). [file HBM-45-e26782-s004.jpg]

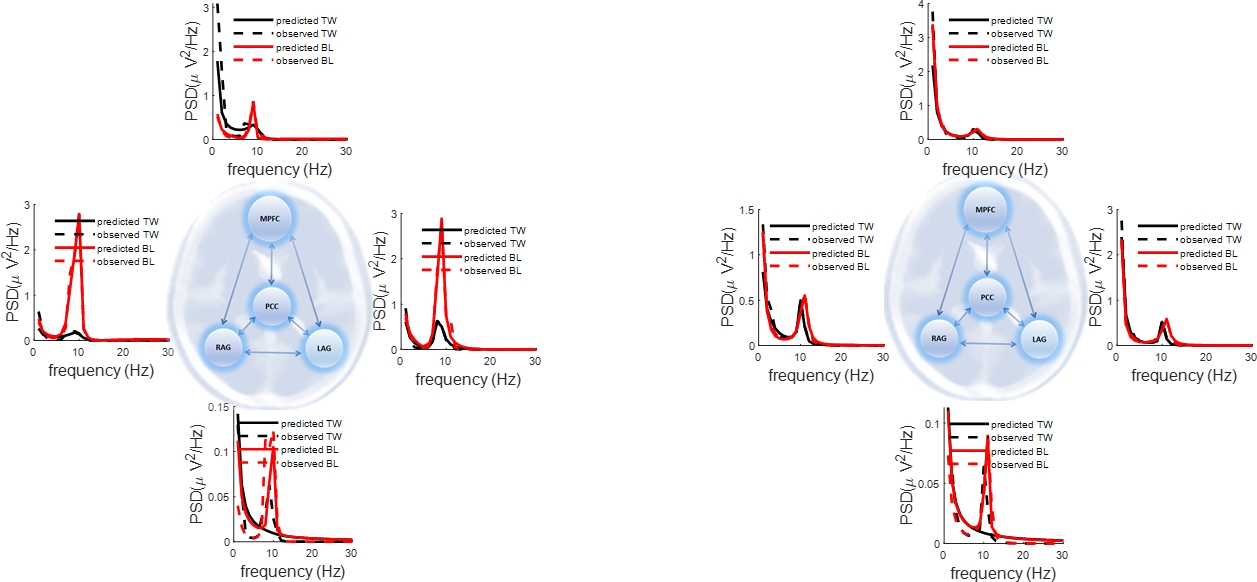

Supplement: Supplementary file 2 — FIGURE 2S. Power spectral densities (PSD) and their associated predicated response by DCM at baseline and after 2 weeks in the eyes open data for the subject 3 (left graphics) and 4 (right graphics). [file HBM-45-e26782-s009.jpg]

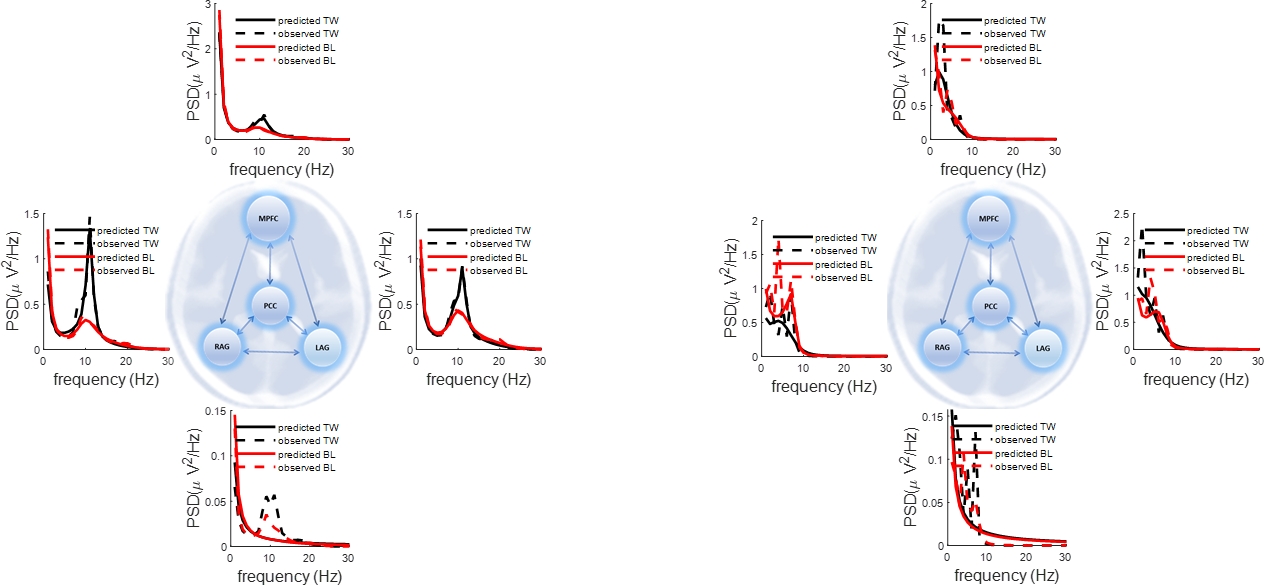

Supplement: Supplementary file 3 — FIGURE 3S. Power spectral densities (PSD) and their associated predicated response by DCM at baseline and after 2 weeks in the eyes open data for the subject 5 (left graphics) and 6 (right graphics). [file HBM-45-e26782-s007.jpg]

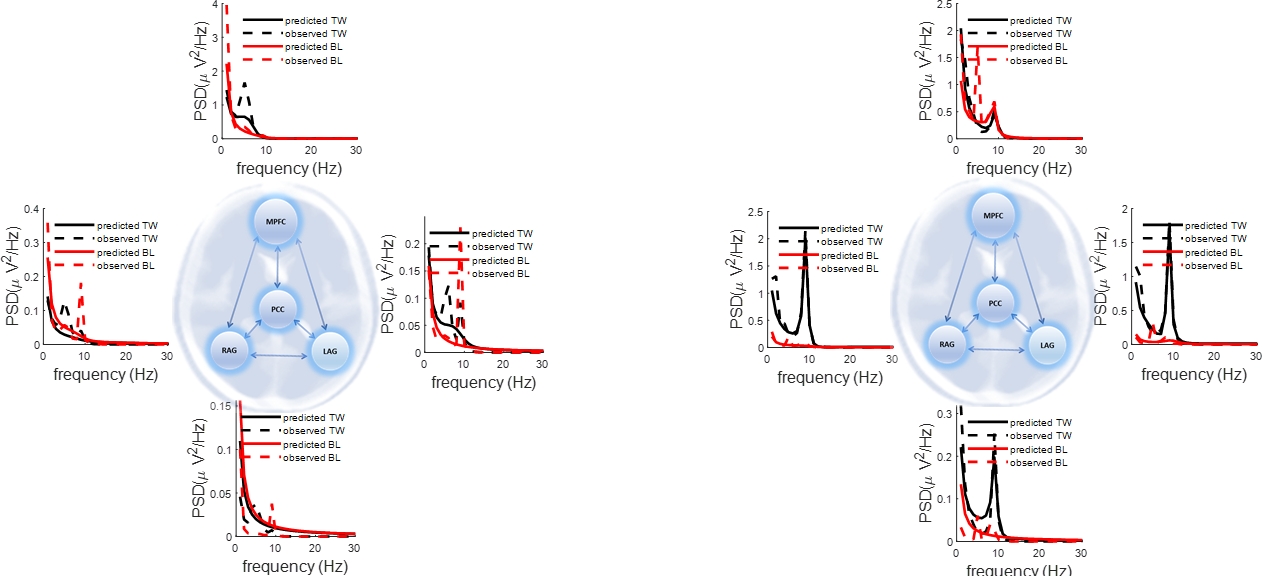

Supplement: Supplementary file 4 — FIGURE 4S. Power spectral densities (PSD) and their associated predicated response by DCM at baseline and after 2 weeks in the eyes open data for the subject 7 (left graphics) and 8 (right graphics). [file HBM-45-e26782-s006.jpg]

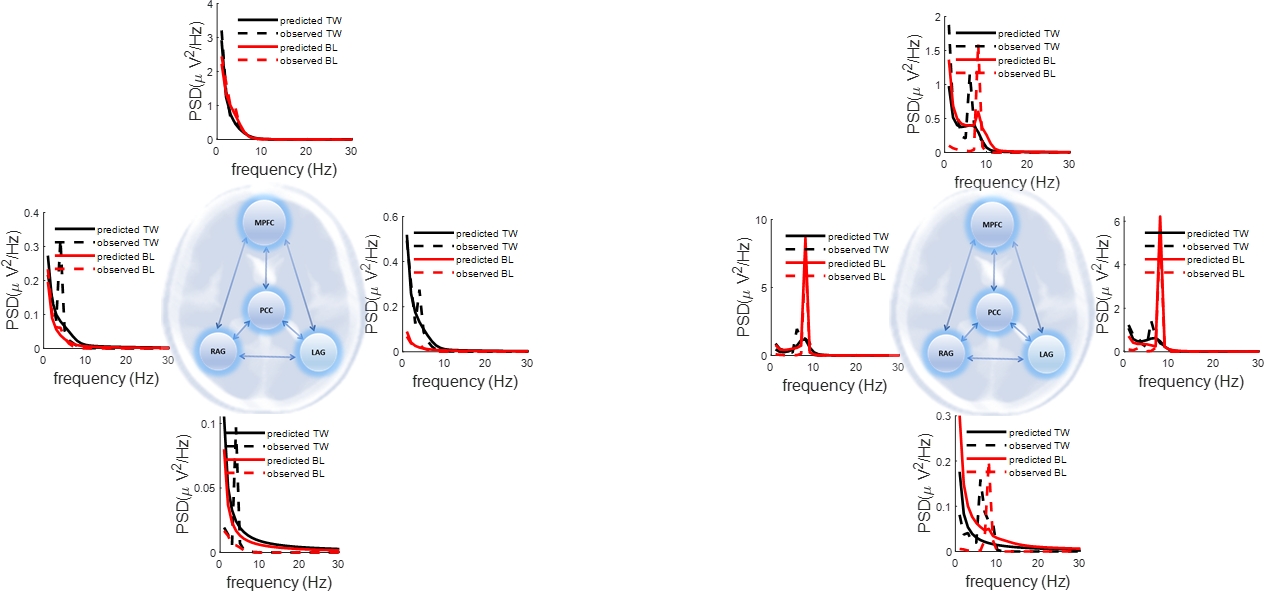

Supplement: Supplementary file 5 — FIGURE 5S. Power spectral densities (PSD) and their associated predicated response by DCM at baseline and after 2 weeks in the eyes open data for the subject 9 (left graphics) and 10 (right graphics). [file HBM-45-e26782-s008.jpg]

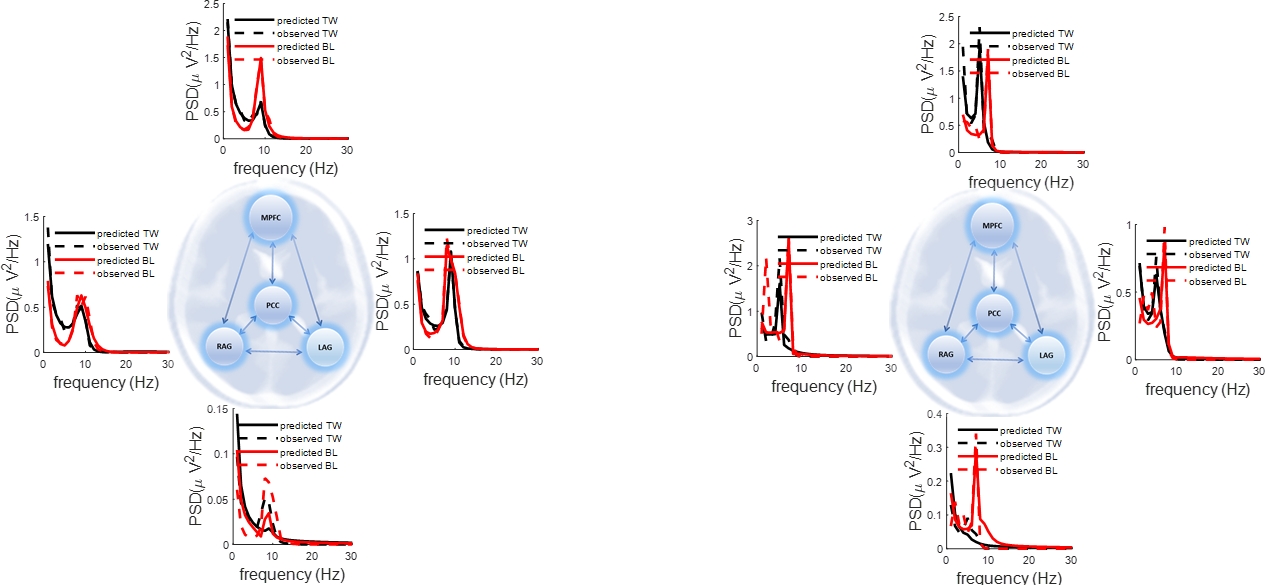

Supplement: Supplementary file 6 — FIGURE 6S. Power spectral densities (PSD) and their associated predicated response by DCM at baseline and after 2 weeks in the eyes open data for the subject 11 (left graphics) and 12 (right graphics). [file HBM-45-e26782-s003.jpg]

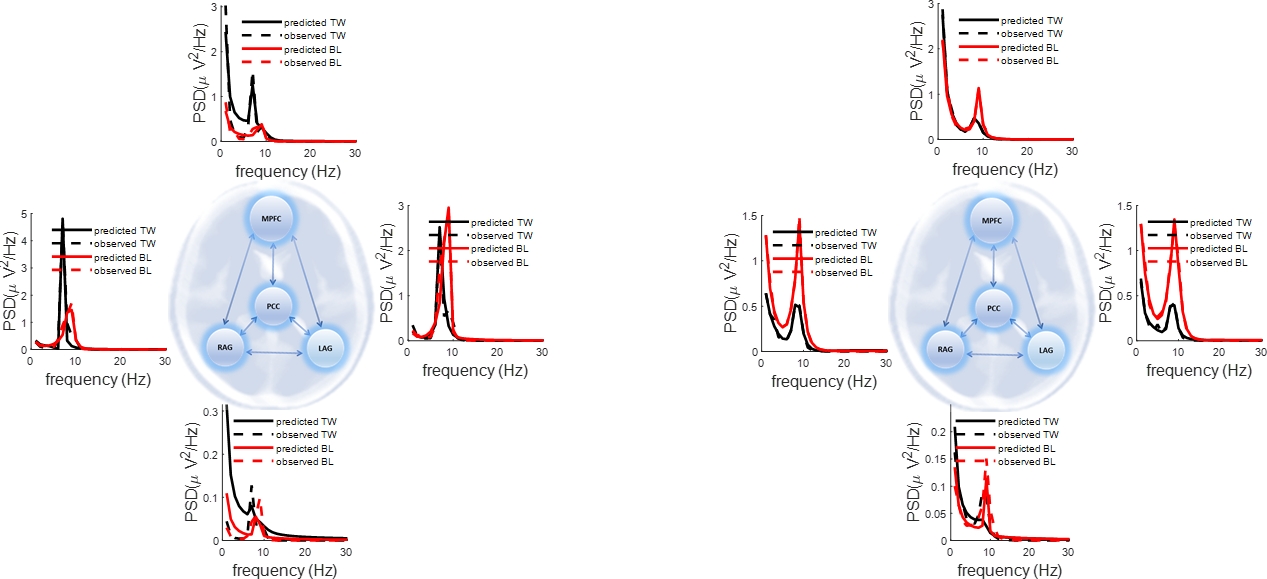

Supplement: Supplementary file 7 — FIGURE 7S. Power spectral densities (PSD) and their associated predicated response by DCM at baseline and after 2 weeks in the eyes open data for the subject 13 (left graphics) and 14 (right graphics). [file HBM-45-e26782-s005.jpg]

Table 1S: the list of the parameters in order of the x axis of the figure 4.


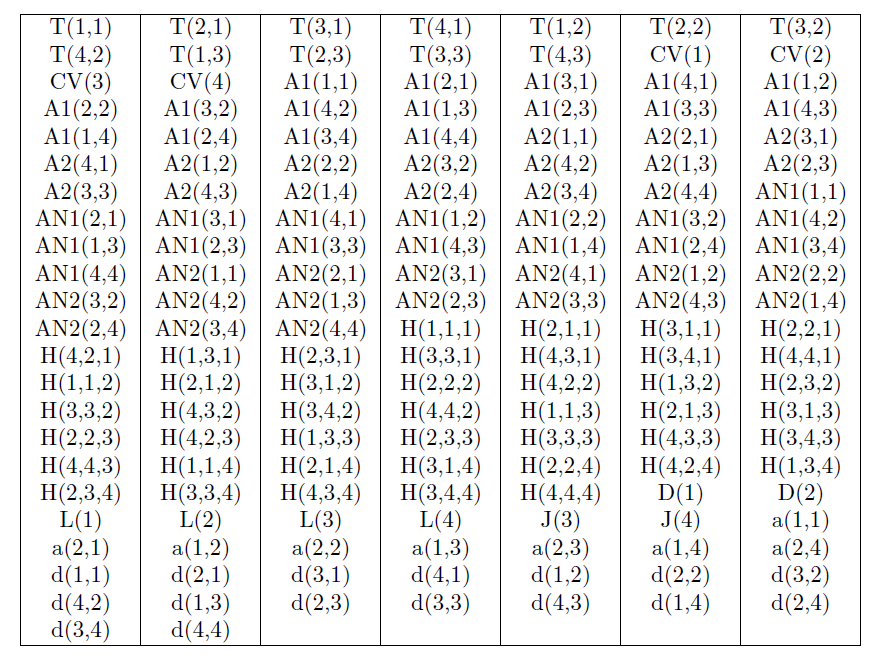

Supplement: Supplementary file 8 — TABLE 1S: The list of the parameters in order of the x axis of the Figure 4. [file HBM-45-e26782-s002.docx]
