# Supplementary material for: Reliability of dynamic causal modelling of resting‐state magnetoencephalography
Source: Hum Brain Mapp. 2024 Jul 11;45(10):e26782. doi: 10.1002/hbm.26782 (PMC11237883; doi:10.1002/hbm.26782)
Supplement: Supplementary file 9 — Supporting information. [file HBM-45-e26782-s001.docx]

The graphics in Figure 1s to 7s are power spectral densities (PSD) and their associated predicated response by DCM at baseline and after two weeks in the eyes open condition, for the same subjects, at the four default mode network sources. Note that differences observed between baseline and re-test data two weeks later are not attributable to the progression of the disease in such a short time but may be related to other factors e.g. plasticity, psychological effects, differential fatigue, measurement noise or movement. Predicted simulated responses for the four-node default model network DCMs suggest that the neuronal model replicates most regions' PSDs well. Note that DCM inversion gives weights or uncertainty to each frequency bin of PSDs, where intuitively low-frequency contents in observed signals may be considered less critical to essential brain rhythms, e.g., alpha, beta, etc. In addition, where a region’s activity (e.g. PCC with maximum alpha peak of 0.05 (𝜇𝑉^2)/𝐻𝑧) is much smaller compared to another region (e.g. LAG with maximum PSD of 2 or 3 (𝜇𝑉^2)/𝐻𝑧), the overall model likelihood and estimated signal-to-noise ratio (precision of the difference between model output and data) may be less sensitive. The list of all parameters in the x axis of the figure 4 are given in the Table 1S.
